# The Penn Medical University

**Published:** 1881-10

**Authors:** 


### THE PENN MEDICAL UNIVERSITY.

"The Historian" of this "University" claims it was a homœopathic institution (though allopathy was taught), because its professors of botany and pathology lectured on homœopathy. Such a claim the Hahnemann Medical College of Chicago, deems absurd. In its latest "announcement" we read:

"Particular attention is called to the fact that in this college and hospital, by the requirements of its special charter, all instruction must be given by practitioners of homœopathy. It is evident that such teaching is preferable, and prevents that mongrelism that must ensue in colleges with mixed schools of practice, or where a majority of the professors have little or no faith in the truth of the homœopathic law."

